# Supplementary material for: Feasibility, Tolerability and Efficacy of Carfilzomib in Combination with Lenalidomide and Dexamethasone in Relapsed Refractory Myeloma Patients: A Retrospective Real-Life Survey of the Sicilian Myeloma Network
Source: J Clin Med. 2019 Jun 19;8(6):877. doi: 10.3390/jcm8060877 (PMC6617295; doi:10.3390/jcm8060877)
Supplement: Supplementary file 1 [file jcm-08-00877-s001.pdf]

Table S1. Comparison of patients' characteristics treated or not with lenalidomide before treatment with KRd in real life.

	Previous Exposure to Lenalidomide (*n* = 53) Group A	No Previous Exposure to Lenalidomide (*n* = 77) Group B	*p*-Value
**Median age, years (range)**	65 (45–86)	64 (33–78)	0.92
**Males, *n* (%)**	30 (57%)	43 (56%)	0.98
**Paraproteins (isotype), *n* (%)**			
Immunoglobulin G	33 (62%)	45 (59%)	0.92
Immunoglobulin A	11 (21%)	13 (17%)	0.88
Immunoglobulin M	1 (2%)	1 (1%)	0.96
Light chain only	8 (15%)	16 (21%)	0.75
Not secretory	0 (0%)	2 (2%)	0.65
FISH			
failed- or not evaluable	28 (53%)	38 (49%)	0.78
Standard risk	15 (28%)	30 (39%)	***0.02***
High-risk	10 (19%)	9 (12%)	***0.02***
ISS stage			
I	36 (68%)	45 (58%)	***0.02***
II	9 (17%)	16 (21%)	***0.02***
III	8 (15%)	16 (21%)	***0.02***
Previous treatments			
Single autologous SCT	15 (28%)	25 (32%)	0.87
Double autologous SCT	17 (32%)	23 (30%)	0.92
Median of prior treatments	2 (1–10)	1 (1–7)	***0.0001***

Table S2. Comparison of patients' characteristics treated or not with pomalidomide before treatment with KRd in real life.

	Previous Exposure to Pomalidomide (*n* = 16)	No Previous Exposure to Pomalidomide (*n* = 114)	*p*-Value
**Median age, years (range)**	68 (52–77)	64 (33–86)	***0.03***
**Males, n (%)**	9 (56%)	64 (56%)	0.89
**Paraproteins (isotype), n (%)**			
Immunoglobulin G	6 (38%)	69 (61%)	***0.03***
Immunoglobulin A	3 (19%)	23 (20%)	0.89
Light chain only	7 (43%)	22 (19%)	***0.02***
FISH			
failed-not evaluable	13 (81%)	85 (75%)	0.89
del13q	0 (0%)	1 (0.9%)	0.91
t4,14 or t 11,14	1 (6%)	9 (8.1%)	0.88
del17p	2 (12%)	3 (26%)	0.72
No alterations identified	0 (0%)	16 (14%)	0.03
ISS stage			
I	9 (56%)	72 (63%)	0.23
II	4 (25%)	21 (18%)	0.22

III	3 (19%)	21 (18%)	0.89
Previous treatment			
Single autologous SCT	6 (38%)	34 (30%)	***0.06***
Double autologous SCT	2 (13%)	38 (33%)	***0.04***
Median of prior treatments	4 (1–10)	2 (1–7)	***0.0001***

Table S3 Univariate and multivariate analysis of PFS and OS in 21 patients who completed at least 18 cycles of KRd.

			**Univariate Analysis**		**Multivariate Analysis**		**Univariate Analysis**		**Multivariate Analysis**	
		n	**PFS @ 24 moNths**	***p*-value**	**HR (95% CI)**	***p*-Value**	**OS @ 24 Months**	***p*-Value**	**HR (95% CI)**	***p*-Value**
**Age**	<=65	7	94.1	0.3			93.7	0.19		
	>65	14	57.6				77.9			
**Gender**	male	11	90.5	0.08			90.5	0.45		
	female	10	57.8				77.8			
**ISS**	I-II	14	8.5	0.33			91.3	0.15		
	III	7	71.4				71.4			
**Prior lines**	1, 2	14	84.1	0.052			95.5	***0.004***		
	more than 2	7	57.1				57.1			
**Previous lenalidomide**	no	4	98.0	0.09			98.0	0.21		
	yes	17	69.4				81.7			
**Previous pomalidomide**	no	20	78	0.62			86.2	0.70		
	yes	1	100				100			
**Previous BMT**	no	6	44.4	***0.01***			77.8	0.33		
	yes	15	90.7				90.4			
**Zoledronic acid exposure**	no	5	75.0	0.48			87.5	0.98		
	yes	16	81.0				86.1			
**Relapse**	biochemical	6	83.3	0.96			83.3	0.64		
	clinical	15	78.0				87.7			
**ClCr**	<30 ml/min	6	33.3	***<0.0001***	22 (2.5-202.3)	***0.006***	33.3	***<0.0001***	na	
	>30ml/min	15	90.4				98.2			
**K Cumulative dose (2 cycles)**	<310mg/mq	7	78.6	0.65			85.4	0.83		
	>310mg/mq	14	88.9				87.5			
**Best response**	<VGPR	8	76.2	0.92			85.7	0.94		
	CR+VGPR	13	80.0				86.7			
